# Community-based specialist palliative care is associated with reduced hospital costs for people with non-cancer conditions during the last year of life

**DOI:** 10.1186/s12904-017-0256-2

**Published:** 2017-12-08

**Authors:** Katrina Spilsbury, Lorna Rosenwax

**Affiliations:** 1 Centre for Population Health Research, Faculty of Health Sciences, Curtin University, Perth, Australia; 2 School of Occupational Therapy and Social Work, Faculty of Health Sciences, Curtin University, GPO Box U1987, Perth, 6845 Australia

**Keywords:** Palliative care, Heart failure, Renal failure, Liver failure, Chronic obstructive pulmonary disease, Cancer, Alzheimer’s disease, Parkinson’s disease, Hospital costs

## Abstract

**Background:**

Community-based palliative care is associated with reduced hospital costs for people dying from cancer. It is unknown if reduced hospital costs are universal across multiple life-limiting conditions amenable to palliative care. The aim of this study was to determine if community-based palliative care provided to people dying from non-cancer conditions was associated with reduced hospital costs in the last year of life and how this compared with people dying from cancer.

**Method:**

A retrospective population-based cohort study of all decedents in Western Australia who died January 2009 to December 2010 from a life-limiting condition considered amenable to palliative care**.** Hospital costs were assigned to each day of the last year of life for each decedent with a zero cost applied to days not in hospital. Day-specific hospital costs averaged over all decedents (cohort averaged) and decedents in hospital only (inpatient averaged) were estimated. Two-part models and generalised linear models were used.

**Results:**

The cohort comprised 12,764 decedents who, combined, spent 451,236 (9.7%) days of the last year of life in hospital. Overall, periods of time receiving community-based specialist palliative care were associated with a 27% decrease from A$112 (A$110-A$114) per decedent per day to $A82 (A$78-A$85) per decedent per day of CA hospital costs. Community-based specialist palliative care was also associated a reduction of inpatient averaged hospital costs of 9% (7%-10%) to A$1030 per hospitalised decedent per day. Hospital cost reductions were observed for decedents with organ failures, chronic obstructive pulmonary disease, Alzheimer’s disease, Parkinson’s disease and cancer but not for motor neurone disease. Cost reductions associated with community-based specialist palliative care were evident 4 months before death for decedents with cancer and by one to 2 months before death for decedents dying from other conditions.

**Conclusion:**

Community-based specialist palliative care was associated with hospital cost reductions across multiple life-limiting conditions.

## Background

Health care at the end of life has been estimated to account for around 10–15% of total health care expenditure in developed countries [1–3]. Health care costs increase sharply as death approaches which has raised concerns about the use of unnecessary medical interventions during the end-of-life phase, although patient social and religious determinants for more aggressive end of life care have been reported [4, 5]. Yet, a recent Italian clinical trial that randomised prostate cancer patients into systematic or on-demand palliative care reported that patients delivered systematic palliative care spent more days in hospice care and received less chemotherapy in the last month of life [6].

In addition to improved patient quality of life measures, specialist palliative care, both hospital-based and community (home)-based, have consistently been associated with reduced health care costs, mostly from a provider viewpoint. A recent systematic review identified 46 studies that compared costs of a palliative care intervention relative to a comparator group with the majority of studies reporting a statistically significant decrease in provider costs for the palliative care intervention [7].

Most of these published studies estimating the cost of palliative care relative to other types of care focused on cancer patients. There are few studies that have compared hospital costs associated with palliative care intervention over the last year of life at a population-level for both cancer and non-cancer conditions. An early US study of over 50,000 Medicare enrolees aged 65 years and over found reduced hospital expenditures in cancer patients who received hospice care but not for non-cancer conditions, however, only univariate analyses were performed [8].

We hypothesised that specialist palliative care provided in the community to people dying from non-cancer conditions would also be associatied with reduced hospital costs in the last year of life as has been demontrated for cancer. The benefits of specialised palliative care for patients with various non-cancer life limiting conditions are now widely reported and include reduced hospitalisation, improved symptom burden and quality of life [9–13]. Despite this, palliative care in non-cancer life limiting conditions is generally considered as under-utilised [14, 15]. In Western Australia (WA), for example, around 70% of people with cancer accessed specialist palliative care in the last year of life compared to 14% of people who died from other conditions considered amenable to palliative care [16].

In Australia, the majority of hospital and palliative care funding is provided by state governments. The WA governament has taken a different approach to other Australian states with the provision of relatively fewer hospital inpatient pallitative care beds and greater funding for community-based specialist palliative care. The aim of this paper was to estimate from a provider perspective, whether receiving community-based specialist palliative was associated with reduced hospital costs in a variety of non-cancer life-limiting conditions considered amenable to palliative care. These findings will provide evidence for service providers and health care planners make more efficient use of the health care resources and to advocate for more equitable access of palliative care across all life-limiting conditions.

## Methods

### Study design

This was a retrospective cohort study of the last year of life of all people who died in WA from 1 January 2009 to 31 December 2010 with a cause of death potentially amenable to receiving palliative care; namely cancers, heart failure, renal failure, liver failure, chronic obstructive pulmonary disease, Alzheimer’s disease, motor neurone disease, Parkinson’s disease, Huntington’s disease and HIV/AIDS. Approvals to conduct this study were granted by Human Research Ethics Committees at the WA Department of Health (#2012/76, 01/02/2013) and Curtin University (53/2012, 06/06/2012).

### Cohort selection

A de-identified linked extraction of death records, hospital records and community-based care records of all decedents spanning the last 2 years of life was obtained from the Data Linkage Branch at the WA Department of Health. Further details on the selection of this cohort and health service use in the last year of life have been reported previously[16, 17]. Briefly, it consisted of 12, 817 people who died during the 2 year study period in WA and who had mention on Part I of their death certificate of one or more of the ten disease conditions considered amenable for palliative care as defined by Rosenwax et al.[18]. When a decedent had more than one of the ten conditions of interest on Part 1 of their death certificate, then the most antecedent condition was assigned as the principal condition.

### Exposure variable: community-based specialist palliative care

The community-based specialist palliative care in this study was provided by Silver Chain WA which supplied over 90% of referred community-based specialist palliative care in WA, although this was mostly restricted to major metropolitan areas [19]. WA is the largest state of Australia (2.5 million km^2^) with a population of 2.6 million that is concentrated in the capital city, Perth, and the south-west corner of the state. A multidisciplinary team of palliative care clinicians and nurses, allied health professionals and volunteers provide home nursing care, counselling, respite options, practical support and links to other services with the aim of enabling people with a life limiting illness to remain at home. A palliative nurse consultancy service was available to residential care facilities where client care is managed around the clock by registered nurses. This service included advice, assessment, staff education and telephone follow-up to meet the needs of specific clients. A palliative care rural telephone advisory service provided specialist palliative care advice to local rural service providers 24 h per day. Client dates of enrolment and disenrollment were used to define periods of time receiving community-based specialist palliative care. It was possible for decedents to have multiple periods of palliative care enrolment if their symptoms were of a relapsing and remitting nature. Clients were discharged from the service after 1 month if they were not available for regular review.

### Outcome measures: day-specific hospital costs

Each day of the last year of life for every decedent was assigned a day number ranging from 1 (365 days before death) to 365 (day of death). A hospital cost was applied to each day of the last year of life for every decedent with days not spent in hospital considered to have incurred zero costs. From these day-specific hospital costs, two cost-based outcome measures were defined. The first was the mean day-specific hospital cost averaged over all decedents whether they were in hospital or not. We refer to this mean day-specific hospital cost as being a cohort averaged (CA) hospital cost. This type of cost estimate is a reflection of both the decision to go to hospital and the actual costs once admitted. The second outcome measure was a mean day-specific hospital cost averaged over only decedents who were in hospital on that particular day. These costs are a reflection of hospital costs once admitted and these are referred to as inpatient averaged (IA) hospital costs.

The Australian Refined-Diagnostic Related Groups (AR-DRGs) system was used to estimate day-specific acute care hospital costs [20]. Component costs for acute episodes of care for each AR-DRG over the study period were obtained from National Hospital Cost Data Collection (NHCDC) reports [21, 22]. Fixed hospital costs included costs of operating room procedures, specialist procedure suites, critical care, pharmacy, pathology, imaging, allied health professionals and emergency department. Fixed costs were considered to have mostly been incurred during the initial period of hospital stays [23]. A daily fixed cost was estimated by fitting the fixed costs to a decreasing exponential distribution where the shape parameter was a function of the actual or national average length of stay such that 99.9% of fixed costs were distributed by the end of the hospital stay. Variable costs included staff salaries and on-costs, hotel costs, supplies and depreciation and were considered to have been incurred at a steady rate over the actual length of stay. Daily fixed and variable costs were summed to generate a day-specific hospital cost per decedent per day. For subacute care hospital stays, the WA average daily cost estimated from the NHCDC Round 15 (2010-2011) report and was assigned as $844 for rehabilitation, $1051 for palliative care, $815 for geriatric evaluation and management, $1162 for psychogeriatric care and $1012 for maintenance [24]. All costs are in Australian dollars ($A) and were adjusted for health inflation and standardised to the 2010-2011 financial year. On 31st December 2011, $A1 was equivalent to $US1 and Euro €0.79. The costs of hospital emergency department presentations were not included in this study.

### Decedent factors

Marital status was classified as partnered or not/unknown. Decedent’s residential locations were used to assign accessibility categories based on the Australian ARIA+ index that takes into account road distance measurements to the nearest service centres and population size [25]. Type of residence was classified as a private residence, residential aged care facility (RACF) or other. Changes in a decedents’ marital status, place of residence, accessibility to services and community-based specialist palliative care status over the last year of life were recorded as time-varying covariates.

Comordity was estimated from hospital inpatient medical records over the last 2 years of life by summing the number of the 31 medical conditions identified by Elixhauser [26] based on algorithms created by Quan [27]. The underlying cause of death was excluded from the calculation of the number of comorbid conditions. Comorbidity for decedents without hospital admissions in the last 2 years of life was estimated from the death certificate.

### Statistical methods

Data were structured so that each decedent had 365 observations representing their last year of life. Each day was assigned as being a day of a hospital stay and/or community-based specialist palliative care or not. Dates were permitted to overlap, for example, a day coded as a hospital stay could also be a community-based specialist palliative care day if it fell between palliative care enrolment and disenrollment service dates. Decedents with incomplete demographic information were excluded from further analysis. Locally weighted scatterplot smoothing was used to produce unadjusted curves of the mean daily hospital costs over the last year of life.

Cohort averaged daily hospital costs were modelled as a mixed discrete-continuous random variable with a mass point at zero. A two part model was fitted with the first part as a binary choice model of being in hospital or not (zero versus non-zero hospital costs) on any 1 day and the second part was conditional on being in hospital that day and used a generalised linear model with a reciprocal link and gamma distribution. The combined prediction was computed as the product of the probability of a hospital admission and the expected value of hospital costs conditional upon a positive cost.

Inpatient averaged mean daily hospital costs were modelled using generalised linear models with inverse Gaussian distribution and inverse quadratic link. Minimisation of the deviance, Akaike and Bayesian information criteria and the modified Park test were used to identify the best fitting distribution and link. Fractional polynomial transformations of variables were used when required. Additional explanatory power of interaction terms was assessed by the difference in model deviance values. Undue influence of individual observations on the model fit were tested and the model fit assessed by plotting Anscombe residuals against log variance. Standard errors were calculated using the clustered sandwich estimator to account for intragroup correlation and the Sidak correction was applied when estimating multiple marginal means. All analysis was performed using Stata 14 (College Station, TX).

## Results

The cohort of decedents with underlying causes of death amenable to palliative care comprised 12,817 individuals as reported previously [16]. After excluding 34 decedents with incomplete demographic information and 19 decedents with Huntington’s disease or HIV/AIDS due to small numbers, there remained 12,764 decedents in this study cohort. Combined, the cohort spent 451,236 (9.7%) days of the last year of life as hospital inpatients for a total hospital cost of A$503 million. Summary characteristic of the cohort are presented in Table 1. The mean and median total hospital costs per decedent in the last year of life and averaged over all decedents were AU$39,369 (SD 41,818) and A$28,182 (IQR 10,732-53,645) respectively. Community-based specialist palliative care was accessed by 3884 (30.4%) decedents during the last year of life and this accounted for 6.6% of days in the last year of life (Table 1). Ten per cent (*n* = 1269) of the cohort were not admitted to hospital in the last year of life.

**Table 1:** Cohort characteristics at death and the crude and adjusted cohort averaged hospital cost estimates by decedent characteristics

Decedent status at time of death (*N* = 12,764 decedents)				Cohort averaged cost per decedent per day (N = 4,658,860 days)					
				% days^b^	Crude A$		Adjusted^a^ A$		
Characteristic		n	%		Mean	SD	Mean	95%CI	*p*-value
Accessed community palliative care	No	8880	69.6	93.4	103	412	112	110-114	ref
	Yes	3884	30.4	6.6	181	438	82	78-85	<0.001
Age group (years)	<60	1640	12.8	12.8	146	521	119	113-125	ref
	60-69	1995	15.6	15.6	127	473	110	105-115	0.011
	70-79	3200	25.1	25.1	114	434	105	102-109	<0.001
	80-89	4208	33.0	33.0	96	363	107	103-110	<0.001
	90+	1721	13.5	13.5	67	281	98	92-104	<0.001
Sex	Male	6910	54.1	54.1	113	434	108	105-110	ref
	Female	5854	45.9	45.9	101	390	108	106-111	0.742
Partnered	No	6826	53.5	48.3	100	392	113	110-116	ref
	Yes	5938	46.5	51.7	115	434	104	102-106	<0.001
Accessibility	Major cities	8769	68.7	69.0	109	413	108	106-110	ref
index	Inner regional	2120	16.6	16.2	100	404	102	97-106	0.009
	Outer regional	1218	9.5	9.3	113	414	113	106-119	0.188
	Remote	425	3.3	3.8	114	416	114	103-126	0.300
	Very remote	232	1.8	1.7	116	534	104	88-119	0.563
Private health insurance	No	8225	64.4	64.4	94	385	101	99-103	ref
	Yes	4539	35.6	35.6	134	462	119	115-122	<0.001
Type of residence	Private	9755	76.4	79.1	119	440	112	110-114	ref
	RACF	2711	21.2	18.2	61	281	81	76-86	<0.001
	Other /NFA	298	2.3	2.7	102	376	110	97-124	0.761
Underlying cause of death	Cancers	7392	57.9	57.9	117	428	112	109-114	ref
	Heart failure	2017	15.8	15.8	92	400	98	93-103	<0.001
	Renal failure	1138	8.9	8.9	133	446	110	104-117	0.660
	COPD	1089	8.5	8.5	95	401	108	101-115	0.295
	Alzheimer’s	605	4.7	4.7	37	216	88	73-103	0.003
	Liver failure	206	1.6	1.6	131	527	98	85-111	0.038
	MND	136	1.1	1.0	77	317	99	79-119	0.202
	Parkinson’s	181	1.4	1.4	48	244	85	70-100	0.001
No. comorbid conditions	None	2526	19.8	19.8	68	307	67	64-70	ref
	One	2834	22.2	22.2	80	354	83	80-86	<0.001
	Two	2344	18.4	18.4	96	387	101	97-105	<0.001
	Three	1745	13.7	13.7	114	428	116	111-121	<0.001
	Four	1222	9.6	9.6	141	495	139	132-146	<0.001
	Five or more	2093	16.4	16.4	182	539	169	163-176	<0.001

*RACF* residential aged care facility, *COPD* chronic obstructive pulmonary disease, *MND* motor neurone disease, *SD* standard deviation, *NFA* no fixed address, *A$* Australian dollars. ^a^Predicted hospital costs from main effects two-part model that included all covariates listed in table plus the closeness to time of death as a 3,3 fractional polynomial transformation of day and the number of previous hospital admissions. ^b^ The percentage of days of the last year of life that decedents spent in each characteristic state. This varies from the percentage at time of death for characteristics which changed over the last year of life

### Cohort averaged (CA) hospital costs

**Fig. 1:**
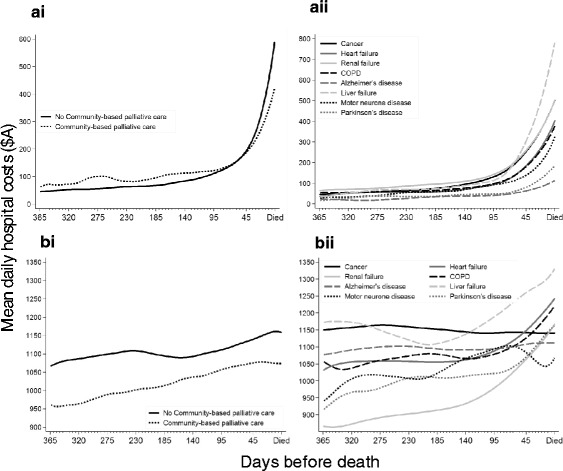
Crude cohort averaged and inpatient averaged day-specific hospital costs by cause of death and community-based palliative care. Locally weighted scatterplot smoothing of day-specific hospital costs over the last year of life as **a** cohort averaged and **b** inpatient averaged and stratified by i) periods of time receiving or not receiving community-based palliative care and ii) principal underlying cause of death

The crude CA hospital cost averaged of the last year of life was A$108 (SD 414) per decedent per day. However, this varied with closeness to time of death and ranged from A$42 (SD 256) 1 year before death to A$602 (SD 714) per decedent per day at time of death. The greatest differences in crude CA hospital costs by community-based specialist palliative care status were observed close to the time of death (Fig. 1Ai). Decedents with liver failure had the highest unadjusted CA hospital costs close to the time of death while those with Alzheimer’s disease and Parkinson’s disease had the lowest (Fig. 1Aii). Higher crude CA hospital costs were also observed in decedents who were younger, male, partnered, had private health insurance, lived in a private residence and had multiple comorbidity (Table 1).

After adjusting for age, comorbidity and other potentially confounding variables in a two part main effects model, community-based specialist palliative care was associated with an average 27% reduction in CA hospital costs over the last year of life. This equated to a A$30 (95%CI 26-34) reduction per decedent per day relative to periods of time not receiving community-based specialist palliative care (Table 1). Decedents dying from heart failure, Alzheimer’s disease and Parkinson’s disease had significantly lower average CA hospital costs relative to decedents with cancer. Adjusted CA hospital costs for decedents with liver failure were A$14 per decedent per day lower than costs for decedents with cancer; a reversal of the earlier crude cost findings. Reduced CA hospital costs were also noted with increasing age at death, having a partner, not having private health insurance, living in a residential aged care facility and having less comorbidity.

**Fig. 2:**
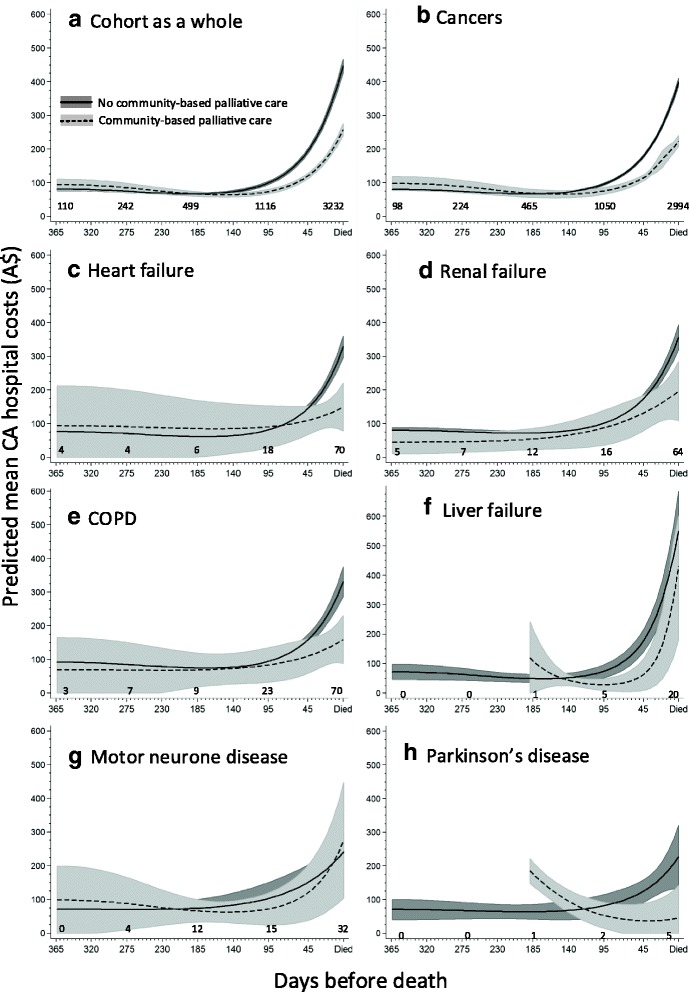
Predicted adjusted cohort averaged day-specific hospital costs by cause of death and community-based palliative care status. Estimated from a two part model that included an interaction term of periods of time receiving community-based palliative care with closeness to time of death and cause of death and adjusted for covariates in Table 1. The number of decedents receiving community-based palliative care on days 1, 90, 180, 270 and 365 before death are indicated on each graph. Graphs were truncated until the time at least one decedent started receiving community-based palliative care and stable estimates could be obtained. The Sidak correction for multiple comparisons was applied. Shading represents 95% confidence intervals. (*N* = 4,658,860 days)

However, a significant three-way interaction was observed between closeness to time of death, cause of death and community-based specialist palliative care which means that a single cost value cannot explain the associations adequately. Averaged over all causes of death, the association of community-based specialist palliative care with reduced CA hospital costs became evident at around 120 days before death (Fig. 2a). When stratified by cause of death, decedents with cancers showed this reduction earlier, at around 140 days before death (Fig. 2b). For decedents with heart failure, renal failure, chronic obstructive pulmonary disease and Parkinson’s disease, significantly reduced CA hospital costs associated with community-based specialist palliative care became apparent around 45 days before death (Fig. 2c to e, h). Decedents with liver failure had reduced CA hospital costs in the fourth to second last months of life with the hospital costs converging around death (Fig. 2f). Decedents with motor neurone disease (MND) showed little difference in CA hospital costs with or without community-based specialist palliative care (Fig. 2g).

### Inpatient averaged (IA) hospital costs

The crude IA hospital cost was A$1114 per hospitalised decedent per day, although this varied with closeness to death and underlying cause of death (Fig. 1Bi and Bii). Being enrolled in community-based specialist palliative care at the time of hospitalisation consistently showed a lower crude IA hospital costs over the last year of life. The crude IA hospital costs stratified by the underlying cause of death showed a more variable pattern. IA hospital costs associated with cancer and Alzheimer’s disease were consistent over the last year of life at around A$1150 and A$1100 per hospitalised decedent/per day respectively regardless of closeness to time of death. In contrast, the three types of organ failure, Parkinson’s disease and COPD all showed increasing IA hospital costs with closeness to time of death. Higher crude IA hospital costs were also observed in decedents who were younger, male, partnered, had private health insurance, lived in a private residence and had multiple comorbidity (Table 2).

**Table 2:** Crude and adjusted inpatient averaged hospital costs stratified by decedent characteristics estimated over inpatient hospital days only

			Inpatient averaged cost per decedent per day (*n* = 451,210 days)				
Decedent characteristics		% of days	Crude A$		Adjusted^a^ A$		
			Mean	SD	Mean	95%CI	p
Receiving community	No	88.5	1120	844	1126	1118-1134	ref
palliative care	Yes	11.5	1064	435	1030	1021-1039	<0.001
Age group (years)	<60	16.6	1166	992	1171	1147-1195	ref
	60-69	17.8	1154	918	1147	1127-1167	0.109
	70-79	26.2	1126	849	1122	1106-1138	0.001
	80-89	30.5	1075	649	1078	1067-1088	<0.001
	90+	9.0	1032	480	1038	1023-1053	<0.001
Sex	Male	56.1	1130	851	1126	1116-1137	ref
	Female	43.9	1093	748	1097	1086-1108	<0.001
Partnered	No	45.9	1091	765	1126	1094-1117	ref
	Yes	54.1	1133	842	1097	1110-1130	0.070
Private health insurance	No	56.4	1106	792	1110	1100-1120	ref
	Yes	43.6	1124	827	1118	1107-1130	0.286
Residence at death	Private residence	86.5	1122	836	1116	1108-1124	ref
	RACF	10.8	1057	568	1099	1079-1118	0.130
	Other care/NFA	2.7	1058	673	1094	1050-1138	0.358
Underlying cause of death	Neoplasms	61.5	1143	779	1139	1130-1148	ref
	Heart failure	13.6	1106	895	1130	1104-1156	0.569
	Renal failure	12.4	982	799	979	950-1007	<0.001
	COPD	7.6	1097	871	1101	1066-1136	0.057
	Alzheimer’s	1.7	1096	471	1133	1101-1165	0.744
	Liver failure	1.8	1221	1123	1164	1090-1238	0.503
	MND	0.8	1061	591	1048	969-1127	0.047
	Parkinson’s	0.7	1032	512	1036	997-1075	<0.001
No. comorbid conditions	None	12.8	1085	636	1069	1057-1082	ref
	One	16.3	1125	766	1106	1090-1123	<0.001
	Two	16.3	1125	768	1116	1100-1132	<0.001
	Three	14.1	1139	816	1134	1115-1153	<0.001
	Four	12.3	1131	919	1140	1114-1166	<0.001
	Five or more	28.2	1094	865	1116	1098-1135	<0.001

*RACF* residential aged care facility, *COPD* chronic obstructive pulmonary disease, *MND* motor neurone disease, *SD* standard deviation, *NFA* no fixed address, *A$* Australian dollars. ^a^Predicted mean daily hospital costs from GLM (inverse Gaussian distribution with an inverse quadratic link) that included all covariates listed in table plus the closeness to time of death represented by day number

After adjusting for other decedent factors associated with IA hospital costs and averaged over the year, IA hospital costs for hospitalised decedents who were receiving community-based specialist palliative care at the time of admission was A$1030 per decedent per day over the last year of life. This was a reduction of A$96 (95%CI 84- 107) per hospitalised decedent per day, equivalent to a 9% (95%CI 7-10) reduction relative to decedents hospitalised during periods of time not receiving community-based specialist palliative care (Table 2). Reduced IA hospital costs were also noted for decedents who were older, female and with less comorbidity.

**Fig. 3:**
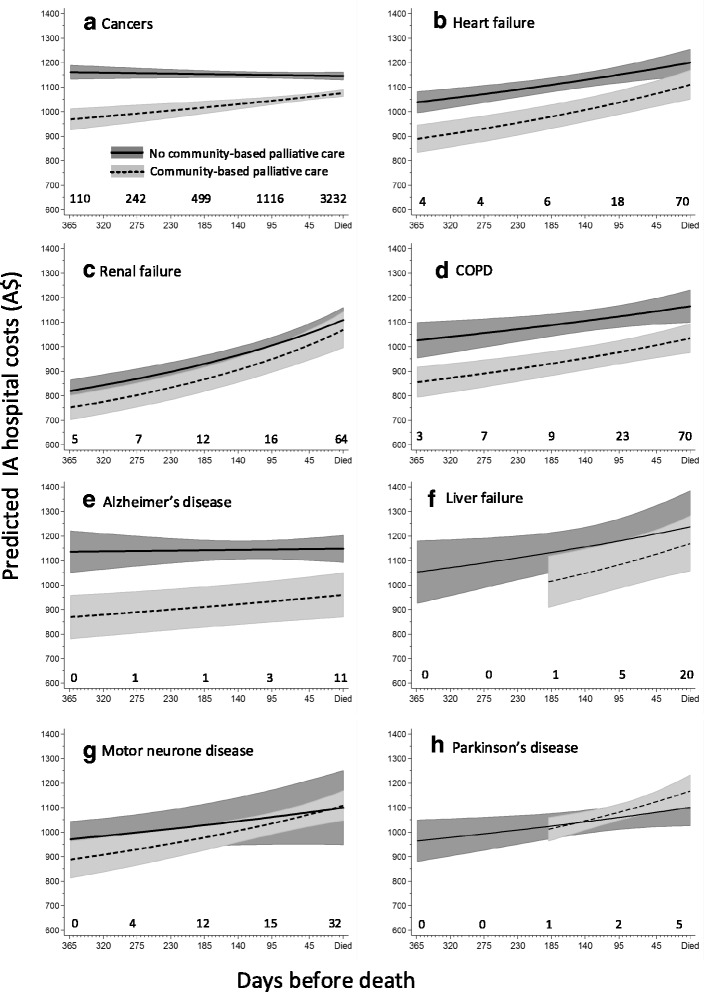
Predicted inpatient averaged day-specific hospital costs by cause of death and community-based palliative care status. Predicted from an inverse Gaussian model adjusted for covariates in Table 2 with cause of death and closeness to time of death as interaction terms with community-based palliative care. Graphs were truncated until the time at least one decedent started receiving community-based palliative care and stable estimates could be obtained. Sidak correction for multiple comparisons applied and number of decedents enrolled in community-based palliative care on selected days indicated. Shading represents 95% confidence intervals. (*N* = 451,210 days)

However, further modelling that included significant interaction terms showed that the reduction in IA hospital costs associated with community-based specialist palliative care varied significantly by the underlying cause of death and closeness to day of death and is best represented graphically (Fig. 3). Periods of time enrolled in community-based specialist palliative care were clearly associated with reduced IA hospital costs for decedents with cancers, heart failure, chronic obstructive pulmonary disease and Alzheimer’s disease (Fig. 3a–e). For decedents with renal failure, Parkinson’s disease and liver failure, the evidence of reduction in IA hospital costs with community-based specialist palliative care was only evident for parts of the last year of life (Fig. 3c–h). Decedents with motor neurone disease showed no significant difference in IA hospital costs with community-based specialist palliative care at any time over the last year of life (Fig. 3g).

## Discussion

Cohort averaged hospital cost reductions associated with community-based specialist palliative care were evident across multiple life-limiting conditions; heart failure, renal failure, chronic obstructive pulmonary disease, liver failure, Parkinson’s disease and cancer but not for motor neurone disease. Inpatient averaged hospital costs associated with community-based specialist palliative care were evident in the organ failures, Alzheimer’s disease, chronic obstructive pulmonary disease and cancer but not in Parkinson’s disease or motor neurone disease. The benefits of specialised palliative care for patients with non-cancer life limiting conditions are also reported to include reduced hospitalisation, improved symptom burden and quality of life [9–13]. Despite this, palliative care in non-cancer life limiting conditions is generally reported as under-utilised [14–16].

We report an average 27% reduction in CA and an average 9% reduction in IA-specific hospital costs during periods of time enrolled in community-based specialist palliative care compared to periods of time not enrolled in this care over the last year of life. This suggests that receipt of community-based specialist palliative care affects both the decision to go to hospital and the hospital costs incurred once admitted, the latter likely a reflection of reduced lengths of hospital stays as we reported previously [28].

In general, the relative reduction in CA hospital costs continued to grow with closeness to time of death for most non-cancer conditions, a pattern observed by others in cancer patients [29, 30]. These relative hospital cost reductions with community-based specialist palliative care were present as early as 4 to 5 months before death in decedents with cancer, but only as early as one or 2 months in most non-cancer conditions. This is partly due to the reduced power to detect cost differences with so few decedents with non-cancer conditions who accessed community-based specialist palliative care. We predict that in a much larger study, earlier hospital cost reductions in non-cancer conditions will be detected.

An exception to the trend of reduced hospital costs with community-based specialist palliative care in non-cancer conditions was seen in decedents with motor neurone disease. This is possibly due to these decedents accessing community-based care through an alternative provider. The Motor Neurone Disease Association in WA is well established and assists clients through support and advisory services, access to specialist clinics, equipment support and accessible transport [31]. However, it is also possible that hospital costs associated with this life-limiting condition are less modifiable or less responsive to community-based specialist palliative care compared to other non-cancer conditions. Further work is needed to clarify this.

The strengths of this study were the large number of decedents from a population base and inclusion of multiple disease conditions amenable to palliative care. Our conclusions are widely generalizable in the Australian context and may also apply internationally where similar models of community-based specialist palliative care are employed. Limitations of this study included possible residual confounding by indication from the lack of clinical detail, particularly for non-hospitalised decedents, and motivations for accessing community-based specialist palliative care. It is likely that patients more accepting of the terminal nature of their condition were likely to receive palliative care and also have a higher threshold before accessing acute care services independent of the palliative care received. We were not able to distinguish between unmeasured patient inclinations and community-based specialist palliative care as drivers of reduced hospital costs in this study. However, a recent randomised controlled trial of cancer patients who were systematically provided with early palliative care resulted in less aggressive end-of-life care when compared to on-demand palliative care; suggesting palliative care itself can be a driver of less hospital use [6].

We also lacked information on the intensity of the palliative care delivered in this study. A dose response has been reported for home based palliative care in an Italian [32] and Canadian [33] setting, where more home nursing was associated with using less acute care services. We did not account for any possible carry over effect of specialist palliative care for decedents who were discharged from the service which may have biased the differences in costs in the week immediately following towards the null. We also presented our findings from a hospital payer perspective only that did not take account of the financial burden to carers and community-based service providers. It has been reported that the largest financial burden for home-based palliative care is the unpaid care costs of patients’ carers [34]. Additional research on the costs of providing community-based specialist palliative care for different life-limiting conditions is also warranted. Our findings are based on data from 2009 to 2010, however, we have no reason to believe that relative hospital costs amongst patient subgroups would have changed significantly over that time.

It would also be naïve to assume that all decedents were willing or able to accept community-based specialist palliative care. While many studies report that most people would prefer to die at home rather than a hospital, there remains patients and carers who feel safer in hospital or have inadequate caring capacity at home [35].

## Conclusion

Community-based palliative care was associated with reduced hospital costs in the last year of life in non-cancer life-limiting medical conditions. Our findings provide further evidence to encourage adoption of community-based specialist palliative care across multiple life-limiting conditions. In an environment of limited health resources, these results should encourage policy makers and service providers to target delivery of palliative care services to all people who could benefit, not just those with cancer.

## Abbreviations

A$: Australian dollar
AR-DRG: Australian Refined-Diagnostic Related Groups
CA: Cohort averaged
IA: Inpatient averaged
NHCDC: National Hospital Cost Data Collection
RACF: Residential aged care facility
WA: Western Australia
